# Transactivation of proto-oncogene c-Myc by hepatitis B virus transactivator MHBs^t167^

**DOI:** 10.3892/ol.2014.2190

**Published:** 2014-05-28

**Authors:** YONG-ZHI LUN, JUN CHENG, QING CHI, XUE-LEI WANG, MENG GAO, LI-DA SUN

**Affiliations:** 1 Liaoning Provincial University Key Laboratory of Biophysics, College of Medicine, Dalian University, Dalian, Liaoning 116622, P.R. China; 2 Institute of Infectious Diseases, Beijing Ditan Hospital, Capital Medical University, Beijing 100015, P.R. China

**Keywords:** MHBs^t167^, transactivation, SV40 early promoter, suppression subtractive hybridization, proto-oncogene c-Myc

## Abstract

C-terminally truncated hepatitis B virus (HBV) middle size surface proteins (MHBs^t^) has been shown to be a transcriptional activator and may be relevant to hepatocarcinogenesis by transactivating gene expression. In the present study, a pcDNA3.1(−)-MHBs^t167^ vector coding for MHBs^t^ truncated at amino acid 167 (MHBs^t167^) was constructed and transfected into the HepG2 hepatoma cell line. mRNA and protein expression of MHBs^t167^ in the cells was detected by reverse transcription-polymerase chain reaction (RT-PCR) and western blot analysis. A cDNA library of genes transactivated by the truncated protein in HepG2 cells was made in pGEM-T Easy using suppression subtractive hybridization. The cDNAs were sequenced and analyzed with BLAST searching against the sequences in GenBank. The results showed that certain sequences, such as that of human proto-oncogene c-Myc, may be involved in tumor development. An expression vector pCAT3/c-Myc containing the chloramphenicol acetyltransferase (CAT) gene under the control of a c-Myc promoter was generated, and the transcriptional transactivating effect of MHBs^t167^ on the c-Myc promoter was investigated by RT-PCR and western blotting. MHBs^t167^ was found to upregulate the transcriptional activity of the promoter, as well as transcription and translation of c-Myc. MHBs^t167^ was also shown to transactivate SV40 immediate early promoter, and transcriptionally transactivate the expression of human c-Myc. These findings provide new directions for studying the biological functions of MHBs^t167^, and for a better understanding of the tumor development mechanisms of HBV infection.

## Introduction

Worldwide, hepatitis B virus (HBV) is an important pathogen causing both acute and chronic liver diseases. Chronic HBV carriers have an increased risk of developing hepatocellular carcinoma (HCC). The mechanism of HBV-related carcinogenesis is poorly understood. Almost all HBV-associated HCCs studied thus far harbor chromosomally integrated HBV DNA (1–3). The integrated HBV DNA can encode two types of transcriptional transactivators: The well studied HBxAg and the preS2 transactivators, large hepatitis B virus surface proteins (LHBs) and C-terminally truncated middle size surface proteins (MHBs^t^) (4–6). The wild-type middle hepatitis B virus surface protein (MHBs) consists of a 55-amino acid (aa) preS2-domain and a 226 aa S-domain. To generate the transactivating forms of MHBs, deletion of at least 87 C-terminal aa is required, and the transcriptional transactivator function is based on the cytoplasmic orientation of the preS2-domain (7). Considerable advances have been made in the fields of transcriptional transactivation of MHBs^t^; however, the molecular basis of MHBs^t^-dependent transcriptional transactivation remains enigmatic. Studies in this area will provide a better understanding of the association between HBV and host hepatocytes, and pave the way for elucidating the pathogenesis of HBV-related HCC.

In the present study, the transactivating potential of MHBs^t^ C-terminally truncated at aa position 167 (MHBs^t167^) on Simian virus (SV40) early promoter was analyzed and, subsequently, genes transactivated by MHBs^t167^ were screened using suppression subtractive hybridization (SSH). SSH was designed to generate a cDNA library that is enriched in differentially expressed sequences and, more importantly, equalized for the number of individual cDNA species, thus allowing the detection of rare transcripts. The full-length genes from the library were searched for homologs in GenBank. Finally, the association between the human proto-oncogene c-Myc and MHBs^t167^ was discussed.

## Materials and methods

### Construction of vectors

For construction of eukaryotic expression vector pcDNA3.1(−)-MHBs^t167^, the MHBs^t167^ fragment was PCR-amplified from pCP10 containing two copies of HBV DNA subtype ayw (GenBank accession: U95551), with the forward primer (5′-GCCGGGCCCATGCAGTGGAATTCCACAAC) containing an *Apa*I site and reverse primer (5′-GGAAAGCTTCTATCCTGGAATTAGAGGACAAAC) containing a *Hin*dIII site. The fragment was inserted into the cloning vector pGEM-T (Promega, Madison, WI, USA), resulting in pGEM-T-MHBs^t167^. An *Apa*I-*Hin*dIII fragment was isolated from the vector and inserted into *Apa*I-*Hin*dIII-digested pcDNA3.1(−) (Invitrogen Life Technologies, Carlsbad, CA, USA), resulting in pcDNA3.1(−)-MHBs^t167^. The pcDNA3.1(−)-MHBs coding for intact HBV middle surface protein was constructed previously in the laboratory (8). To construct the reporter vector pCAT3-c-Myc, the promoter of human c-Myc was amplified from the genomic DNA of the HepG2 human HCC cell line (HBsAg-negative) by PCR with a pair of forward (5′-GGTACCATCCTCTCTCGCTAATCTCC) and reverse (5′-AGATCTATGGG CAGAATAGCCTCCCC) primers containing a *Kpn*I and a *Bgl*II site, respectively. The promoter fragment was inserted into pGEM-T, yielding pGEM-T-c-Myc. A *Kpn*I-*Bgl*II fragment was isolated from the plasmid and inserted at the *Kpn*I-*Bgl*II sites of pCAT3-basic (promoterless; Promega), resulting in the pCAT3-c-Myc reporter vector. DL2000 DNA marker (Takara Biotechnology (Dalian) Co., Ltd, Dalian, China) was used as a DNA molecular weight marker. All the vectors were sequenced and digested with corresponding restriction enzymes to confirm the sequence accuracy.

### Cell culture and transient transfection

HepG2 cells were purchased from the Cell Resource Center, Shanghai Institutes for Biological Sciences, Chinese Academy of Sciences (Shanghai, China) and were cultivated in Dulbecco’s modified Eagle’s medium (Invitrogen Life Technologies) containing 100 IU penicillin and 100 μg streptomycin per milliliter, supplemented with 10% (v/v) heat-inactivated fetal bovine serum (Hyclone Laboratories, Inc., Logan, UT, USA), at 37°C in 5% CO_2_ and 90% relative humidity. The cells were seeded on the day before transfection at a density of 8×10^5^ cells per 35-mm dish and reached 50% confluence at the time of transfection. All transfections were performed with FuGene^®^ 6 Transfection Reagent (Roche Applied Science, Indianapolis, IN, USA) according to the manufacturer’s instructions. The medium was changed 5 h after transfection and cells were harvested 40–48 h following transfection. All transfections and assays were repeated independently three times in triplicate.

### Detection of MHBs^t167^ expression

mRNA from HepG2 cells transfected with pcDNA3.1(−)-MHBs^t167^ and pcDNA3.1(−) was isolated using a QuickPrep Micro mRNA purification kit (Amersham Biosciences, Little Chalfont, UK), and cDNA was reverse-transcribed from the mRNA. MHBs^t167^ expression was detected by reverse transcription-polymerase chain reaction (RT-PCR) with MHBs^t167^-specific primers by using 35 amplification cycles, and by western blotting using lysates of the HepG2 cells. The extracts were boiled for 5 min and separated by SDS-polyacrylamide gel electrophoresis (SDS-PAGE), and then transferred to nitrocellulose membranes (Pierce Biotechnology, Inc., Rockford, IL, USA). The membranes were reacted with anti-pre-S2 mouse anti-human monoclonal antibody and HRP-labeled goat anti-mouse polyclonal IgG as the primary and secondary antibodies, respectively and then with a SuperSignal West Pico Chemiluminescent Substrate working solution (Pierce Protein Biology Products, Thermo Fisher Scientific, Inc., Rockford, IL, USA) according to the manufacturer’s instructions. The immunoreactive bands were visualized after exposure to X-ray film.

### Detecting the effect of MHBs^t167^ on SV40 early promoter and c-Myc promoter

To detect the effect of MHBs^t167^ on the SV40 promoter, various quantities of reporter vector pCAT3-promoter (Promega) containing CAT reporter gene controlled by the SV40 immediate early promoter element were transiently transfected into HepG2 cells. Co-transfections were made with pcDNA3.1(−)-MHBs^t167^ (2.0 μg) + pCAT3-promoter (0.4 μg) as the test group, and pcDNA3.1(−) (2.0 μg) + pCAT3-promoter (0.4 μg) and pcDNA3.1(−)-MHBs (2.0 μg) + pCAT3-promoter (0.4 μg) as the control groups, respectively. To detect the effect of MHBs^t167^ on the c-Myc promoter, various quantities of pCAT3-c-Myc were transiently transfected into HepG2 cells. The co-transfections were made with 1.0 μg reporter vector pCAT3-c-Myc and 0.5, 1.0, 1.5 and 2.0 μg effector vector pcDNA3.1(−)-MHBs^t167^. The relative CAT activity was measured using enzyme-linked immunosorbent assay according to the manufacturer’s instructions (CAT ELISA kit; Roche Applied Science).

### Generation and analysis of a subtracted cDNA library

SSH was performed with the PCR-Select™ cDNA subtraction kit (Clontech Laboratories, Inc., Mountain View, CA, USA) according to the manufacturer’s instructions. In brief, 2.0 μg of poly A+ mRNA, each from the pcDNA3.1(−)-MHBs^t167^ tester group and the pcDNA3.1(−) driver group was subjected to cDNA synthesis, respectively. Following restriction with *Rsa*I, small sizes of cDNAs were obtained. The tester cDNAs were then subdivided into two parts, ligated with the specific adaptor 1 and adaptor 2, respectively. After two subtractive hybridization reactions and two suppression PCR amplifications, differentially expressed cDNAs were selectively amplified. Subsequently, the second PCR products were used as templates for PCR amplification of G3PDH (a housekeeping gene) at 18, 23, 28, 33 cycles, respectively, to analyze subtraction efficiency. The second PCR products were directly purified using the Wizard^®^ PCR-Preps DNA Purification system (Promega), and inserted into pGEM-T Easy (Promega) to construct the subtracted library. Colony PCRs were conducted to confirm that the size of the cDNA inserts ranged between 200 and 1,000 bp by using T7/SP6 specific primers localized in pGEM-T Easy. Following DNA sequencing of the positive colonies, nucleotide homology searches were performed using the BLAST program at NCBI (http://blast.st-va.ncbi.nlm.nih.gov/Blast.cgi).

### Detecting the effect of MHBs^t167^ on c-Myc expression

To detect the effect of MHBs^t167^ on c-Myc mRNA, total RNA was extracted from HepG2 cells transiently transfected by pcDNA3.1(−) and pcDNA3.1(−)-MHBs^t167^ using TRIzol reagent (Invitrogen Life Technologies) according to the manufacturer’s instructions, and was used for RT-PCR. The following primers (sense, 5′-TTCGGGTAGTGGAAAACCAG and antisense, 5′-CAGCAGCTCGAATTTCTTC) were used to amplify the c-Myc cDNA. β-actin specific primers (sense, 5′-TGACGGGGTCACCCACACTGTGCCCATCTA and antisense, 5′-CTAGAAGCATTTGCGGTGGACGATGGAGGG) was used as internal reference. To detect the effect of MHBs^t167^ on c-Myc protein, total soluble proteins were extracted in radioimmunoprecipitation assay buffer (Pierce Biotechnology, Inc.) from the transfected HepG2 cells and separated on 12.5% SDS-PAGE gels for immunoblotting assay (prepared in house, Institute of Infectious Diseases, Beijing Ditan Hospital, Capital Medical University, Beijing, China). The expression of c-Myc was probed by mouse monoclonal antibody against human c-Myc derived from a cell line from American Type Culture Collection, Manassas, VA, USA. Mouse anti-human monoclonal β-actin antibody (Santa Cruz Biotechnology, Inc., Santa Cruz, CA, USA) was used as internal reference.

## Results

### Transient expression of MHBs^t167^ in HepG2 cells

**Figure 1:**
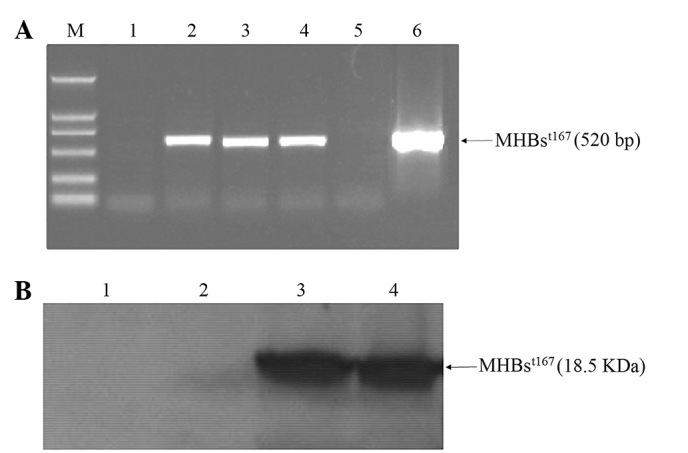
Transient expression of MHBs^t167^ in HepG2 cells. (A) Products of reverse transcription-polymerase chain reaction amplification of MHBs^t167^ mRNA. Lane 1, mRNA from HepG2 cells; lanes 2–4, mRNA from HepG2 cells transfected with pcDNA3.1(−)-MHBs^t167^; lane 5, mRNA from HepG2 cells transfected with pcDNA3.1(−); lane 6, pcDNA3.1(−)-MHBs^t167^ vector positive control; M, DL2000 DNA marker. (B) Western blot analysis of MHBs^t^ protein. Lane 1, lysates from HepG2 cells; lane 2, lysates from HepG2 cells transfected with pcDNA3.1(−); lanes 3 and 4, lysates from HepG2 cells transfected with pcDNA3.1(−)-MHBs^t167^. MHBs^t167^, hepatitis B virus middle size surface protein C-terminally truncated at amino acid position 167; M, molecular weight marker.

Digestion of recombinant vector pcDNA3.1(−)-MHBs^t167^ with *Apa*I/*Hin*dIII, *Eco*RI, *Xba*I and *Xho*I yielded the expected bands (data not shown). DNA sequencing results indicated that the recombinant vector contained HBV DNA fragment encoding the truncated middle surface protein in-frame and the sequence was completely correct. MHBs^t167^ mRNA and protein expression in HepG2 cells was successfully detected by RT-PCR (Fig. 1A) and western blotting (Fig. 1B), respectively.

### Transactivation of MHBs^t167^ on SV40 immediate early promoter

**Figure 2:**
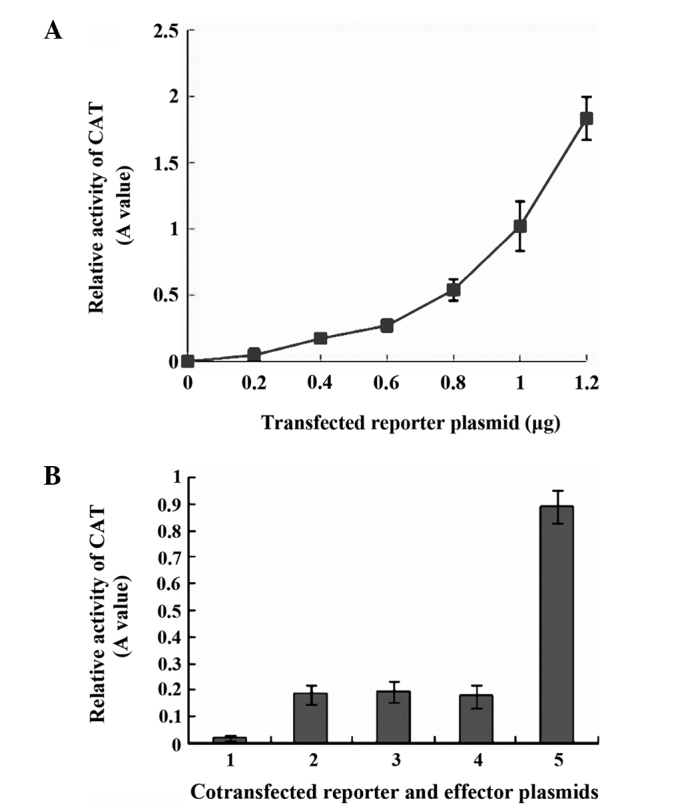
Transactivation of MHBs^t167^ on SV40 early promoter in HepG2 cells. (A) The association between the quantity of reporter vector and the relative activity of CAT. (B) Transactivation of SV40 promoter element by MHBs^t167^. Lane 1, 0.4 μg of pCAT3-basic (promoterless); lane 2, 0.4 μg of pCAT3-promoter (SV40 promoter); lane 3, 0.4 μg of pCAT3-promoter and 2.0 μg of pcDNA3.1(−); lane 4, 0.4 μg of pCAT3-promoter and 2.0 μg of pcDNA3.1(−)-MHBs; lane 5, 0.4 μg of pCAT3-promoter and 2.0 μg of pcDNA3.1(−)-MHBs^t167^. The standard deviation is shown in the diagram. MHBs^t167^, hepatitis B virus middle size surface protein C-terminally truncated at amino acid position 167; SV40, Simian virus 40; CAT, chloramphenicol acetyltransferase.

To determine the sensitivity of the kit for CAT measurement, we first evaluated the CAT activities in total cell lysates of HepG2 cells transfected with different quantities of pCAT3-promoter. The results showed that CAT gene expression exhibited an approximately linear association with the quantity of reporter vector used in transfection (Fig. 2A). Subsequently, 0.4 μg of reporter vector was selected to be used in the transfection experiments, so that it was easy to detect the reporter activity with room for further increase if a greater quantity of reporter vector was used. In the transient co-transfection assays, CAT gene expression from the pCAT3-promoter was ~4.5-fold higher following co-transfection with pcDNA3.1(−)-MHBs^t167^ compared with that after co-transfection with pcDNA3.1(−) or pcDNA3.1(−)-MHBs. The marked increase in CAT gene expression may be attributed to the transactivating effect of the truncated HBV MHBs^t167^ on the SV40 early promoter element, leading to the observed increase in CAT expression, whereas the intact MHBs was not transactive (Fig. 2B).

### Analysis of the cDNA subtracted library transactivated by MHBs^t167^

**Figure 3:**
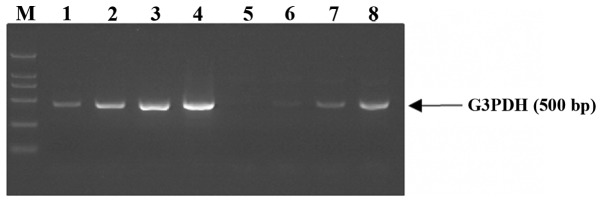
Analysis of subtracted cDNA library (reduction of G3PDH abundance showed high subtraction efficiency). Lanes 1–4 (unsubtracted) and lanes 5–8 (subtracted): secondary polymerase chain reaction product. Lanes 1 and 5, 18 cycles; lanes 2 and 6, 23 cycles; lanes 3 and 7, 28 cycles; lanes 4 and 8, 33 cycles. M, molecular weight marker.

To gain a general view of the genes which may be involved in the pathogenesis of HBV, genes that were upregulated in HepG2 cells expressing MHBs^t167^ were identified by the generation of a subtracted cDNA library. Subtraction efficiency analysis showed that PCR products of the housekeeping gene G3PDH in the unsubtracted library were obviously visible after 18 cycles; however, 28 cycles were required in the subtracted library (Fig. 3), indicating that the abundance of non-differentially expressed genes was effectively reduced and the subtraction method had a high subtraction efficiency. Using SSH, a total of 94 positive clones were obtained. These clones were prescreened by using PCR amplification to ensure that they had different inserts before sequencing. Among these clones, 77 contained inserts of 200–1,000 bp. A total of 50 clones from the cDNA library were randomly chosen and sequenced, and their nucleotide sequence homology searches were performed using the BLAST program at NCBI. The analysis results showed that there were 22 coding sequences, of which 18 were known and 4 were unknown genes. Some of the proteins coded by these genes have been shown to be involved in cell cycle regulation, cell apoptosis, signal transduction pathways and tumor development. Notably, the cell proto-oncogene c-Myc was up-regulated by MHBs^t167^. A summary of the data is presented in Table I.

### MHBs^t167^ upregulates the c-Myc promoter activity

**Figure 4:**
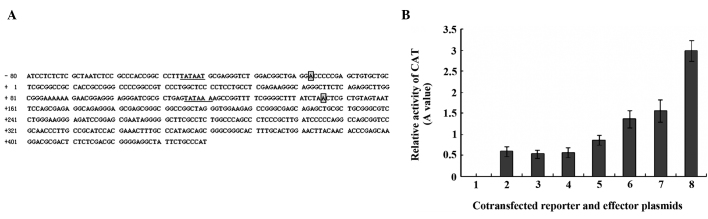
(A) Nucleotide sequence of human oncogene c-Myc promoter. The classical ‘TATA’ boxes are underlined and ‘A’ denotes the start sites of c-Myc promoters 1 and 2, respectively. The first nucleotide of exon 1 acts as +1. (B) Transactivation of human oncogene c-Myc promoter element by MHBs^t167^ in HepG2 cells in a dose-dependent manner. Lane 1, 1.0 μg of pCAT3-basic (promoterless); lane 2, 1.0 μg of pCAT3-promoter (positive control); lane 3, 1.0 μg of pCAT3-c-Myc; lane 4, 1.0 μg of pCAT3-c-Myc and 1.0 μg of pcDNA3.1(−); lanes 5–8, 1.0 μg of pCAT3-c-Myc and 0.5, 1.0, 1.5 and 2.0 μg pcDNA3.1(−)-MHBs^t167^, respectively. The standard deviation is shown in the diagram. MHBst^167,^ hepatitis B virus middle size surface protein C-terminally truncated at amino acid position 167; CAT, chloramphenicol acetyltransferase.

To examine the relationship between MHBs^t167^ and c-Myc, the effect of MHBs^t167^ on the promoter activity of c-Myc was investigated. The promoter activity was evaluated using a CAT assay in HepG2 cells transfected with pcDNA3.1(−)-MHBs^t167^. As shown in Fig. 4, transiently expressed MHBs^t167^ was found to markedly increase the promoter activity of c-Myc in HepG2 cells in a dose-dependent manner. The results suggested that MHBs^t167^ protein could transactivate c-Myc expression by transcriptionally activating its promoter element.

### MHBs^t167^ upregulates the c-Myc expression

**Figure 5:**
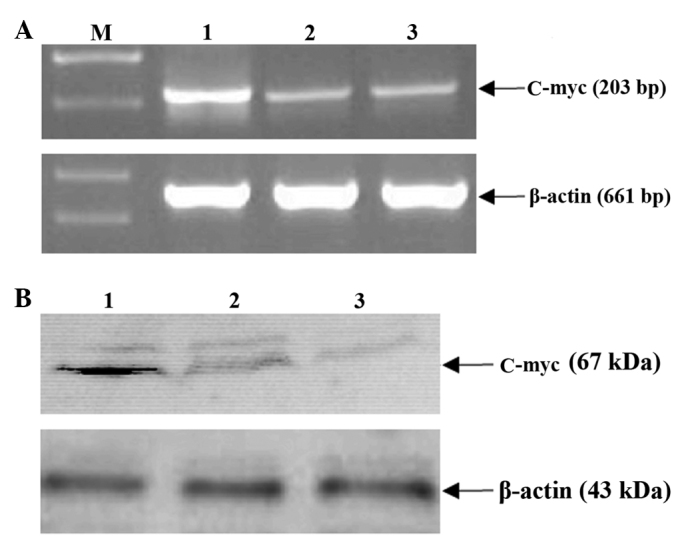
(A) Reverse transcription-polymerase chain reaction and (B) western blot analysis of proto-oncogene c-Myc expression in HepG2 cells transfected with different vectors. Lane 1, HepG2 cells transfected with vector pcDNA3.1-MHBs^t167^; lane 2, HepG2 cells transfected with empty vector pcDNA3.1(−); lane 3, untransfected HepG2 cells. MHBst^167,^ hepatitis B virus middle size surface protein C-terminally truncated at amino acid position 167; M, molecular weight marker.

To further elucidate the mechanisms of MHBs^t167^ on c-Myc expression at the transcription and translation levels, the effect of MHBs^t167^ on expression of the gene was investigated. As shown in Fig. 5A, level of mRNA of c-Myc markedly increased following transient transfection with pcDNA3.1(−)-MHBs^t167^. The western blot analysis indicated that the expression of the gene was low in the control groups, whereas in the experiment group, its expression was markedly enhanced (Fig. 5B). These results indicated that MHBs^t167^ could transactivate the expression of c-Myc at both the transcription and translation levels.

## Discussion

The molecular mechanism of HBV-related carcinogenesis is poorly understood. A possibility could be activation of the expression of cellular genes involved in cell growth regulation by viral proteins. For example, HBx has been shown to act as a transcriptional transactivator, stimulating various viral and cellular enhancer and promoter elements (9). Transactivating functions have also been attributed to viral proteins translated from 3′-truncated HBV preS/S sequences that are frequently found in human HCCs (10). Lauer *et al* found there are three hydrophobic regions in the S-domain, located at MHBs residues 62–78, 135–53 and 224–281, respectively, which are separated by two highly hydrophilic regions (at MHBs residues 79–134 and 154–223). The authors showed that if truncation occurred beyond the third hydrophobic region with the first hydrophobic region being complete, the truncated protein acquired transactivation functions (11). MHBst-encoding sequences are found in numerous integrates subcloned from HBV-associated HCC, and previous studies showed there was truncated-form S protein in the circulation of patients with chronic hepatitis B virus infection (12,13).

To evaluate the putative relevance of MHBs^t167^ in the process in HBV-associated HCC development, a detailed analysis of MHBs^t167^ activator function is of great biological significance. In the present study, the authors demonstrated that MHBs^t167^ was successfully expressed in the transiently transfected HepG2 cells. In the co-transfection experiments, the relative CAT expression in the cells transfected with pcDNA3.1(−)-MHBs^t167^ + pCAT3-promoter was approximately 4.5-fold higher than that with pcDNA3.1(−) + pCAT3-promoter or pcDNA3.1(−)-MHBs + pCAT3-promoter. This indicated that MHBs^t167^ had a significant transactivating function on the SV40 early promoter, leading to the observed increase in the expression of the downstream gene CAT, while the intact MHBs did not show transactivation. This suggests that HBV MHBs^t167^ transiently expressed in HepG2 cells retains its biological activity in transcriptional activation, which is consistent with previous reports (14).

To gain further insights into the genes transactivated by MHBs^t167^, SSH was used to clone the genes transactivated by MHBs^t167^. Sequencing of the genes obtained from the subtracted library revealed 22 different coding sequences, of which18 were known and 4 were unknown genes.

The genes with known functions can be divided into five groups, namely genes related to cell transcription and protein synthesis, cell energy and substance metabolism, the formation mechanism of hepatic fibrosis, cell signal transduction and apoptosis, and tumor development (15). Notably, upregulated expression of proto-oncogene c-Myc was observed. Yuen *et al* demonstrated that 74% of HCC tissues had a high level of c-Myc expression (16). Overexpression of c-Myc has been implicated in liver regeneration and hepatocarcinogenesis. It is also an indicator of malignant potential and poor prognosis (17). The biological significance of c-Myc gene upregulation by the truncated middle surface protein of HBV in human hepatocellular carcinoma, however, has not been confirmed.

To further elucidate the regulatory mechanisms of MHBs^t167^ on c-Myc expression, a reporter vector pCAT3-c-Myc was generated where the CAT gene was placed under the control of the c-Myc promoter, which contains the partly 5′-flanking region and the majority of the exon 1 regions of the c-Myc gene (18). The upregulated expression of proto-oncogene c-Myc in HepG2 cells has been confirmed by cell transient transfection at the mRNA and protein levels. It is reasonable to believe that the transformation effect of MHBs^t167^ is involved in the upregulation of the expression of proto-oncogene c-Myc.

In conclusion, the present study analyzed the transactivator function of MHBs^t167^ and constructed a subtracted cDNA library of genes transactivated by MHBs^t167^. Furthermore, it was confirmed that MHBs^t167^ could transactivate the expression of c-Myc at the transcriptional and translational levels. These findings provide new insights into the biological functions of MHBs^t167^ and new directions to elucidate the hepatocarcinogenesis mechanisms of HBV infection.

## Figures and Tables

**Table I:** Sequence analysis of 42 clones isolated from subtracted cDNA library transactivated by MHBs^t167^.

GenBank accession	Gene description	Number of clones	Homology (%)
NM_001402	Homo sapiens eukaryotic translation elongation factor 1 alpha 1 (EEF1A1)	8	98
NM_001025	Homo sapiens ribosomal protein S23 (RPS23)	6	100
NM_212482	Homo sapiens fibronectin 1 (FN1)	4	100
NM_000477	Homo sapiens albumin (ALB)	3	99
NM_005004	Homo sapiens NADH dehydrogenase (ubiquinone) 1 beta subcomplex, 8, 19kDa (NDUFB8)	3	100
NM_000014	Homo sapiens alpha-2-macroglobulin (A2M)	2	100
NM_000300	Homo sapiens phospholipase A2, group IIA (PLA2G2A)	2	100
NM_001354	Homo sapiens aldo-keto reductase family 1, member C2 (AKR1C2)	2	96
NM_002970	Homo sapiens spermidine/spermine N1-acetyltransferase (SSAT)	2	100
NM_002467	Homo sapiens v-myc avian myelocytomatosis viral oncogene homolog (MYC)	2	98
NM_001032281	Homo sapiens tissue factor pathway inhibitor (TFPI)	1	99
NM_002128	Homo sapiens high mobility group box 1 (HMGB1)	1	100
NM_000126	Homo sapiens electron-transfer-flavoprotein, alpha polypeptide (ETFA)	1	99
NM_000482	Homo sapiens apolipoprotein A-IV (APOA4)	1	99
NM_012073	Homo sapiens chaperonin containing TCP1, subunit 5 (CCT5)	1	99
NM_000687	Homo sapiens adenosylhomocysteinase (AHCY)	1	99
NM_000582	Homo sapiens secreted phosphoprotein 1 (SPP1)	1	95
NM_005141	Homo sapiens fibrinogen beta chain (FGB)	1	100

MHBs^t167^, hepatitis B virus middle size surface protein C-terminally truncated at amino acid position 167.
